# Extended Toxicity, Genotoxicity, and Mutagenicity of Combination of pBudK-coVEGF-coANG and pBudK-coGDNF Plasmids in Preclinical Trials

**DOI:** 10.3390/biomedicines13051223

**Published:** 2025-05-18

**Authors:** Igor V. Samatoshenkov, Alexander M. Aimaletdinov, Elena Y. Zakirova, Egan L. Kalmykov, Rustam Khodzhibaev, Yulia M. Samatoshenkova, Ilnur M. Ganiev, Marat S. Kadyrov, Yana O. Mukhamedshina

**Affiliations:** 1 Medizinische Hochschule Brandenburg Theodor Fontane, 14770 Brandenburg, Germany; 2 Department of Vascular and Endovascular Surgery, Brandenburg University Clinic, 14770 Brandenburg, Germany; kalmykove@yahoo.com (E.L.K.); khodzhibaev@yahoo.de (R.K.); 3 Institute of Fundamental Medicine and Biology, Kazan (Volga Region) Federal University, 420008 Kazan, Russia; allekss1982@mail.ru (A.M.A.); lenahamzina@yandex.ru (E.Y.Z.); ilnurgm-vgora@mail.ru (I.M.G.); yana.k-z-n@mail.ru (Y.O.M.); 4 SAHI Republican Clinical Hospital of the Ministry of Health of the Republic of Tatarstan, 420138 Kazan, Russia; julia.samatoshenkova@mail.ru; 5 Limited Liability Company “Angiolife”, 420015 Kazan, Russia; forts75@mail.ru; 6 Department of Histology, Cytology and Embryology, Kazan State Medical University, 420012 Kazan, Russia; 7 Academy of Sciences of the Republic of Tatarstan, 420013 Kazan, Russia

**Keywords:** genetic therapy, ischemic disease, plasmid, glial-derived neurotrophic factor, vascular endothelial growth factor, angiogenin

## Abstract

Chronic lower limb ischemia is a debilitating condition, particularly prevalent among elderly patients and individuals ineligible for revascularization procedures. Gene therapy aimed at promoting therapeutic angiogenesis presents a promising alternative treatment strategy. **Objectives:** This study evaluated the preclinical safety of a gene therapy drug composed of the plasmids pBudK-coVEGF-coANG and pBudK-coGDNF in laboratory animals. Safety assessment followed a single intramuscular injection at a dose 30 times higher than the proposed therapeutic level. **Methods:** Acute toxicity was monitored over a 24-h period. Genotoxicity was assessed using the micronucleus test at doses of 200, 1000, and 5000 μg/kg. Bone marrow cytology was analyzed to detect hematopoietic toxicity. Delayed toxicity was evaluated over a two-week recovery period. **Results:** No signs of acute toxicity were observed, even at the highest dose. The micronucleus test revealed no genotoxic effects, with no significant increase in micronucleated polychromatic erythrocytes compared to control groups. Bone marrow erythroblast parameters remained within normal physiological ranges. Additionally, no delayed adverse effects were detected during the recovery period. **Conclusions:** The gene therapy drug demonstrated a favorable preclinical safety profile, exhibiting no evidence of toxicity or genotoxicity, even at substantially elevated doses. These findings support the continued development of this therapy as a potential treatment for chronic lower limb ischemia in patients who are not candidates for surgical intervention.

## 1. Introduction

Peripheral arterial disease (PAD) of the lower extremities, primarily caused by atherosclerosis, is recognized by the World Health Organization as one of the most disabling forms of cardiovascular disease [1]. In its advanced stages, the condition leads to chronic lower extremity ischemia (CLI), often resulting in pain, tissue damage, and an increased risk of limb amputation. Over the past two decades, several experimental approaches, including cell- and gene-based therapies, have been investigated to restore perfusion in ischemic tissues, although current clinically available treatments remain largely limited to endovascular procedures and surgical bypass, which are not always feasible in elderly patients or those with diffuse, multilevel arterial involvement [2].

Gene therapy offers a promising alternative for such patients by enabling the localized induction of therapeutic angiogenesis. Although approximately ten gene therapy products have been approved globally for various indications [3], none have yet received regulatory approval for the treatment of chronic lower limb ischemia. Notably, gene therapy has not been recommended in the current clinical guidelines of the European Society for Vascular Surgery [4].

Among the limited examples of gene therapy applications in PAD, Neovasculgen^®^—a plasmid DNA drug encoding human *VEGF165*—was approved in the Russian Federation in 2011 for the treatment of CLI of atherosclerotic origin. Clinical studies, including a five-year follow-up, showed that early administration of Neovasculgen^®^ can slow ischemic progression and reduce the likelihood of amputation [5]. Similarly, Collategene, a hepatocyte growth factor gene therapy approved conditionally in Japan in 2019, showed initial promise in promoting ulcer healing in patients with PAD, with a favorable safety profile apart from localized injection-site reactions [6].

Despite these advancements, concerns remain regarding potential risks associated with gene therapy, including off-target angiogenesis and tumor promotion in predisposed individuals [4]. Thus, the development of novel gene therapy strategies that combine angiogenic efficacy with demonstrated safety remains a high priority.

In response to this unmet need, we developed an innovative gene therapy formulation consisting of two highly purified plasmids: pBudK-coVEGF-coANG and pBudK-coGDNF. This combination uniquely encodes three therapeutic factors: vascular endothelial growth factor (*VEGF*), angiogenin (*ANG*), and glial cell line-derived neurotrophic factor (*GDNF*). *VEGF* is a key regulator of endothelial cell proliferation and neovascularization [7,8,9,10]; *ANG* is among the most potent known inducers of blood vessel formation [11]; and *GDNF* contributes to neurovascular regeneration, epithelial barrier restoration, and tissue repair [12,13,14]. Notably, the regulatory interplay between *VEGF* and *GDNF* offers a novel approach to stimulate both vascular and neuronal recovery in ischemic tissue.

This represents the first gene therapy strategy to combine angiogenic and neurotrophic factors in a single therapeutic platform, with the aim of enhancing microvascular growth while simultaneously supporting nerve regeneration and tissue stability. The current study presents the results of a comprehensive preclinical safety assessment of this gene therapy product in rodents. The primary objectives were to determine the maximum tolerated dose, evaluate potential acute toxic effects within 24 h, and assess delayed toxicity during a two-week follow-up after a single intramuscular injection of high-dose plasmids.

## 2. Methods and Materials

### 2.1. Infrastructure and Set-Up

This study was conducted in accordance with the ethical standards outlined by *Basic and Clinical Pharmacology and Toxicology* for experimental research [15]. Toxicity and genotoxicity evaluations were performed in collaboration with the Laboratory of Biological Testing and the Testing Center of the Shemyakin–Ovchinnikov Institute of Bioorganic Chemistry, Russian Academy of Sciences. All procedures were complied with the *Institute Program for the Care and Use of Laboratory Animals* and were carried out in an AAALAC-accredited facility. Animal care, handling, and monitoring were performed by qualified personnel following standard veterinary guidelines.

### 2.2. Composition of the Novel Compound

In this study, we utilized the pBudCE4.1 vector, which is designed for the concurrent expression of two genes. The vector features a CMV immediate early promoter (human cytomegalovirus) and a human elongation factor 1α subunit to facilitate the independent expression of the two genes.

To enhance the expression efficiency of the *VEGF*, *ANG*, and *GDNF* genes, we employed the OptimumGene algorithm. This algorithm optimizes various factors influencing gene expression, such as codon usage, GC content, CpG dinucleotide presence, mRNA secondary structure, and potential cloning hindrances (e.g., restriction sites and premature polyadenylation). Codon optimization aimed to increase the codon adaptation index (CAI) to 0.95, improving the translational efficiency without altering the amino acid sequences of the proteins encoded by these genes. The optimization process also minimized regions with high GC content, which can enhance mRNA stability, and removed potential cis-acting elements. The resistance gene sequence for zeocin was replaced with the kanamycin resistance gene, and the V5-His and myc-His tag sequences (3127–3195 bp and 719–782 bp, respectively) were removed. The resulting vector was named pBudK.

The novel gene therapy drug consists of a mixture of highly purified plasmids pBudK-coVEGF-coANG and pBudK-coGDNF. These plasmids carry human genes encoding *VEGF*, angiogenin (*ANG*), and glial-derived neurotrophic factor (*GDNF*). Plasmid DNA (pDNA) was quantified by agarose gel electrophoresis and confirmed to be >95% in supercoiled form with an endotoxin level of 0.03 U/mg pDNA in lyophilized form (Supplementary Materials Figure S1). The plasmids were reconstituted in 60 µL of 0.9% solution, and the formulation was administered via intramuscular injection using 2.5 mL syringes (STK Group LLC, Orel, Russia).

### 2.3. Animals

- Maximum Tolerated Dose (MTD) of the Drug

The MTD determination of the new drug was performed on male mice obtained from the nursery for laboratory animal “Pushchino”, which is a branch of the Shemyakin–Ovchinnikov Institute of Bioorganic Chemistry under the Russian Academy of Sciences. The mice were 5 weeks old at the time of testing. The animal breeder provided data from the latest animal health monitoring, which confirmed their SPF status (specific pathogen-free). The animals underwent an examination upon arrival. For this experiment, animals without any indication of health abnormalities were selected (a clinical examination was performed). The animals were grouped according to the randomization principle, using body weight as a criterion, to ensure that the average body weight of the animals on the first day of administration was not statistically different between the groups. The animals were kept in regulated environmental conditions, with a temperature range of 20–24 °C, relative humidity of 30–55%, a 12 h lighting cycle, and a room air volume change rate of 10 times per hour. In each animal room, the temperature and humidity were continuously and automatically monitored by the Eksis Visual Lab system (Praktik-NTs, Moscow, Russia). Wood chips (J. Rettenmaier & Söhne GmbH + Co KG, Rosenberg, Germany) served as the bedding material for rodents, ensuring a dust-free environment. The bedding was autoclaved, and the laboratory performed routine microbiological contamination tests. The standard rodent pellet diet was provided ad libitum in a food hopper on a wire top. Specially prepared Milli-RO (Millipore) water was given ad libitum in standard autoclaved drinking bottles with steel spout-type caps. The preparation of the water ensures that there is no contamination that could affect the test results. Periodic laboratory analysis was carried out on drinking water to detect contamination. Feed consumption was measured before dose administration (day 0–1) and on study days 1–2, 4–5, 7–8, 14–15, and 21–22. Feed consumption of each animal was individually quantified by weighing the feed grate at the start and end of approximately 24 h.

The animals were divided into 2 groups of 5 animals each. Doses for administration were prepared immediately before injection as a carrier solution—sterile water for injection (“Production Pharmaceutical Company Renewal”, Novosibirsk, Russia). The preparation for intramuscular injection was based on the dose value in µg/kg and the volume administered [16]. The first dose was 5000 µg/kg (2500 µg/kg of each plasmid), and the maximum dose was 20,000 µg/kg (10,000 µg/kg of each plasmid). The volume of administration was 4 mL/kg. Syringes 0.5 mL with integrated needle (0.5 mL/cc with 29 G 0.33 mm × 12.7 mm, SFM) were used for administration. At the end of this study (day 22), a routine euthanasia of all animals was performed. The animals were placed in a CO_2_ chamber until they showed signs of death, at which point a complete necropsy was performed.

### 2.4. Toxicity of the Drug

An extended toxicity study of a single dose of the new drug was conducted in rats. The rats, acquired from the Pushchino Laboratory Animal Nursery at 4–5 weeks of age, were acclimated for approximately 3 weeks in the animal facility at the Laboratory of Biological Testing, Shemyakin–Ovchinnikov Institute of Bioorganic Chemistry before being assigned to the experimental groups. Animals considered unsuitable for this study were excluded prior to group formation. The rats were held in a barrier zone of two corridors of the enclosure (barrier zone 1 of the Laboratory of Biological Testing) with automatic cycles of day and night and a ventilation rate of 12 full air volume changes per hour. The animals were kept in accordance with the standards defined in Directive 2010/63/EU on the protection of animals used for scientific purposes. The animals received ad libitum the Laboratory Autoclavable Rodent Diet Spezialdiaten GmbH, Soest, Germany. Autoclaved wood chip bedding (LIGNOCEL BK 8/15, Rosenberg, Germany) was used. Filtered tap water was provided ad libitum in standard drinking bottles. The average temperature in the vivarium ranged from 21 °C to 23 °C, while the average relative humidity was 30% to 58%. An EVL system automatically monitored the temperature and humidity of each room continuously.

Sprague-Dawley (SD) rats were divided into 3 groups. Each group consisted of 13 males and 13 females: control group 1 (0 mg/kg of drug), experimental group 2 (0.2 mg/kg of drug), and group 3 (0.6 mg/kg of drug). Therefore, the new drug was administered to the animals 10 times the clinical dose (0.2 mg/kg) and 30 times the clinical dose (0.6 mg/kg). The doses were administered intramuscularly by a single injection into the femoris quadriceps muscle of the left limb. The volume administered for each group was 0.5 mL/kg body weight (50 µL/100 g body weight). The individual injection volume was determined on the basis of the last body weight value for the selection of the correct dose in mg/kg. For intramuscular injection, a 1 mL syringe with integrated needle was used. The administration of doses to all animals was carried out at approximately the same time each morning (09:00–12:00). The day of dosing was designated as day 1 for the animal enrolled in this study.

Eight males and females from each group were sacrificed on day 2 of this study (24 h after induction); the remaining animals were sacrificed 14 days after induction.

All animals were examined twice daily for indications of mortality and dying animals. A thorough clinical evaluation was performed immediately after dosing and then weekly during the withdrawal period.

Body weight was measured on days 1, 2, 3, 8, and 14 of this study, while feed intake was recorded the day before administration, 24 h after administration, and for the periods between days 2 and 8 and between days 8 and 14 of this study.

At the end of the in-life phase of this study, the animals were sacrificed by anesthesia, followed by a final blood collection for clinical pathological evaluation, including hematology and serum biochemistry analysis. Anesthesia was administered by intramuscular injection of a mixture (80–100 µL/animal) of Telazol^®^ (Zoetis, Girona, Spain) and Xyla^®^ (Interchemie werken “De Adelaar”, Püünsi, Estonia). EDTA blood samples (PanEco, Moscow, Russia) were examined using veterinary software on a Mythic 18 automated hematology analyzer (C2 DIAGNOSTICS SA, Montpellier, France). Serum was analyzed using Randox Laboratories Ltd. reagents on a SAPPHIRE 400 automated biochemical analyzer (Prestige 24i, Tokyo, Japan). Chloride, sodium, and potassium ions were measured with ion-selective electrodes on an EX-D analyzer (JOKOH Co, Ltd., Tokyo, Japan). Glucose was measured in whole blood using the glucose-oxidase electrode method on a Sattelite Express^®^ glucometer (ELTA, Moscow, Russia).

After performing an autopsy on the bodies, the organs were weighed. The tissues of five males and females from groups 1 and 3, were sacrificed on day 2, and five males and females from groups 1 and 3 were examined microscopically. To perform this, tissues were fixed in 10% formalin (Tatkhimpharmpreparaty, Kazan, Russian), embedded in paraffin, and then stained with hematoxylin and eosin (Tatkhimpharmpreparaty, Kazan, Russian) before being studied under a microscope.

### 2.5. Genotoxicity and Mutagenicity

This study was carried out on male and female ICR outbred mice. Animals aged 7–8 weeks and weighing 27–30 g were used for this experiment. The animals were housed under standard vivarium conditions. The vivarium maintained a regimen with automatic alternation of day and night. They received autoclaved feed from the Laboratory Rodent Diet and filtered water. The water was given ad libitum in standard drinking bottles.

The selection of doses for preclinical trials of gene therapy drugs is based on the rate of effective plasmid doses extrapolated to clinical use. According to the Scientific and Regulatory Considerations in the Safety Evaluation of Gene Therapy Products in Preclinical Studies Considerations for Preclinical Safety, three doses should be used in preclinical safety trials of vector gene drugs, the lower dose can match the effective dose, and the larger dose exceeds the maximum clinically possible dose by 10 times.

The drug was injected intramuscularly, the method recommended in the clinic. The recommended volume for intramuscular injection in rodents is 50–60 µL into the muscle, which is 2 mL/kg for mice. Doses were prepared as a combined solution of two plasmids in sterile water for injection. For intramuscular injection, 0.5 mL of syringes with integrated needle (0.5 mL/cc with 29 G 0.33 mm × 12.7 mm, SFM) were used.

The experimental animals were divided into 5 groups. Animals in the first group received the drug intramuscularly into the quadriceps muscle of the thigh at a dose of 200 µg g/kg. The animals of the second group received the drug in a dose of 1000 µg/kg. Mice in the third group received the drug at a dose of 5000 µg/kg. The fourth group was a negative control group. These animals were injected with injection water, which is the solvent for the gene drug. The solvent and the test drug were injected twice at 3-day intervals. The animals in the fifth group were positive controls. They received a single intraperitoneal injection of cyclophosphamide at a dose of 50 mg/kg in a volume of 10 mL/kg.

**Figure 1:**
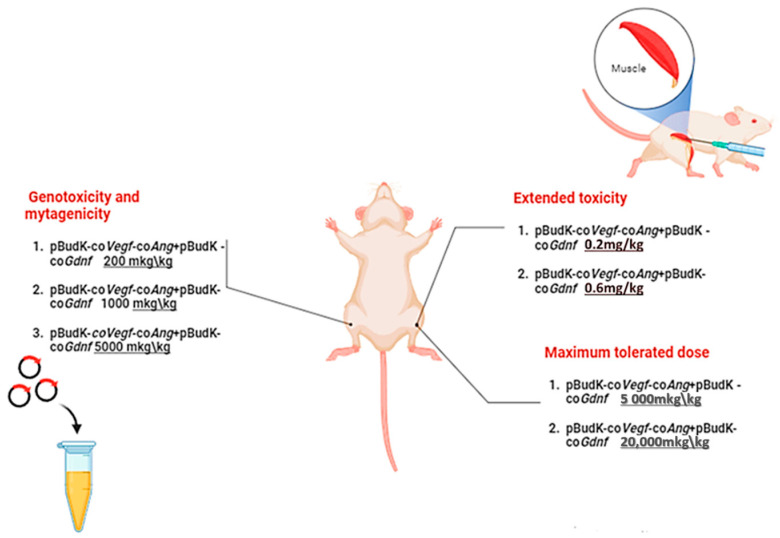
Experimental design and group assignment. Intramuscular injection of plasmids.

Twenty-four hours after injection, the animals were sacrificed in a CO_2_ chamber. Immediately after euthanasia, mice were aspirated with bone marrow from the thigh bone and smears were prepared, followed by Romanowsky and May–Grünwald fixation and staining. Using a light microscope, 4000 immature (polychromatic) erythrocytes (PCEs) were analyzed in a smear for the presence of micronuclei (the frequency of micronucleated polychromatic erythrocytes) and 500 erythrocytes with differentiation into polychromatic and mature (normochromic) to determine the ratio between them. The general scheme of the experiments is in Figure 1.

### 2.6. Statistical Processing

R language for statistical computing was used for the data analysis. Illustrations were created using package “ggplot2”. Quantitative variables were represented with minimum (min), maximum (max), median (M), first (Q1) and third (Q3) quartiles, average/mean (Mean), and standard deviation (SD). Statistical comparisons were carried out using Mann–Whitney U test on log10-transformed values in order to obtain median ratios and their non-parametric 95% confidence intervals. Reported values were backtransformed. Statistical significance cutoff was selected as *p* < 0.05. Correction for multiple comparisons was carried out using Benjamini–Hochberg method.

## 3. Results

### 3.1. MTD

To assess the safety profile of the test plasmid-based drug, mice were administered a cumulative dose of 5000 µg/kg, which exceeds the estimated potential clinical dose by approximately 250-fold. No adverse effects were observed at this dose level. Subsequently, a maximum dose of 20,000 µg/kg was administered to a separate group of mice. This higher dose was also well tolerated, with no toxic effects noted.

Following administration of 5000 µg/kg, animals appeared clinically normal, with the exception of transient vocalization during the second injection. In the group receiving 20,000 µg/kg, a temporary reduction in activity was observed in all animals approximately 30 min post-injection. Additionally, two animals exhibited vocalization during the second portion of dosing. All observed behavioral signs resolved spontaneously within one hour.

Body weight measurements taken before and after treatment showed no notable changes in either dose group. Feed consumption remained stable throughout the observation period. Necropsy findings revealed no macroscopic abnormalities attributable to the test compound.

These findings indicate that the plasmid-based drug is well tolerated in mice at doses up to 20,000 µg/kg, with only mild and transient clinical signs observed at the highest dose. No systemic toxicity or organ-level pathology was detected.

### 3.2. Extended Toxicity

#### 3.2.1. Clinical Observations and Body Weight

No mortality or severe clinical signs were observed in any treatment group. All animals survived until the scheduled euthanasia. During both the acute phase and the 14-day observation period, no clinical abnormalities related to the test compound were observed in either sex. Body weight and weight gain remained unaffected by the treatment. No statistically notable differences were observed between the treated groups and the control group receiving water for injection. Similarly, food consumption remained consistent across all groups, with no significant treatment-related differences.

#### 3.2.2. Organ Weights

**Figure 2:**
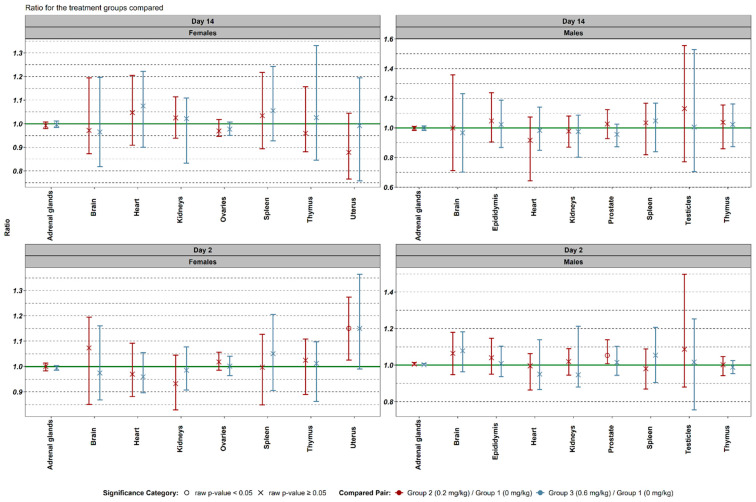
Comparison of the data on organ weight in relation to body weight between different experimental groups. Log10 transformed values were used to obtain median ratios and their non-parametric 95% confidence intervals. Reported values were backtransformed.

On day 2, an important increase in relative uterus weight was observed in female rats administered 0.2 mg/kg (group 2) compared to controls. A similar trend was noted at 0.6 mg/kg (group 3), though it did not reach statistical significance. In males, the 0.2 mg/kg group exhibited a significant increase in relative prostate weight, whereas this effect was not observed in the 0.6 mg/kg group (Figure 2).

#### 3.2.3. Hematological Parameters

- Females (Day 2):

Both doses (0.2 and 0.6 mg/kg) resulted in a significant increase in absolute and relative granulocyte content (2.00× increase with overlapping 95% CIs indicating statistical significance). A significant reduction in relative lymphocyte content was observed in both groups (ratios of 0.87 and 0.88, respectively). Plateletcrit and absolute platelet counts were significantly reduced in both groups. A slight but significant increase in mean platelet volume (MPV) was noted in group 2 but not in group 3. Non-significant trends included a decrease in absolute lymphocyte content and an increase in relative monocyte content.

- Males (Day 2):

Group 3 (0.6 mg/kg) exhibited a significant increase in relative granulocyte content (1.22×, 95% CI: 1.02–1.67). A significant decrease in platelet count and plateletcrit was also noted in this group. Group 2 (0.2 mg/kg) showed a reduction in RDW-SD (red cell distribution width–standard deviation).

- Females (Day 14):

No significant hematological changes were observed in females.

**Figure 3:**
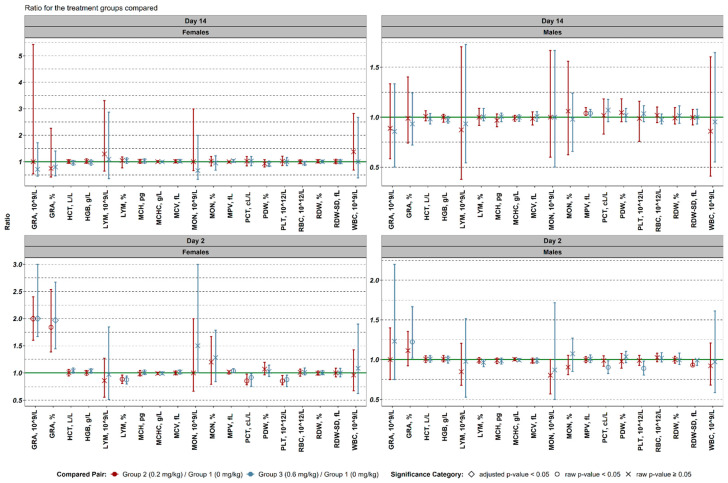
Comparison of the hematological data between different experimental groups. Log10 transformed values were used to obtain median ratios and their non-parametric 95% confidence intervals. Reported values were backtransformed.

- Males (Day 14):

In males, mean platelet volume was significantly elevated in both treatment groups (1.04× vs. control), indicating a potential persistent effect on platelet morphology or turnover (Figure 3).

These hematological findings suggest that the test compound induces sex- and dose-specific changes, particularly in granulocyte activation and platelet parameters, indicative of potential innate immune stimulation and transient myelosuppressive effects.

#### 3.2.4. Biochemical Parameters

- Females (Day 2):

Although biochemical data showed variability across both treatment groups, no statistically significant changes were detected.

- Males (Day 2):

Group 3 (0.6 mg/kg) showed a notable reduction in aspartate aminotransferase (AST) levels (0.85×; 95% CI: 0.71–0.97). Alkaline phosphatase (ALP) levels were significantly decreased in both group 2 (0.79×) and group 3 (0.78×), suggesting possible liver, biliary, or bone metabolism modulation.

**Figure 4:**
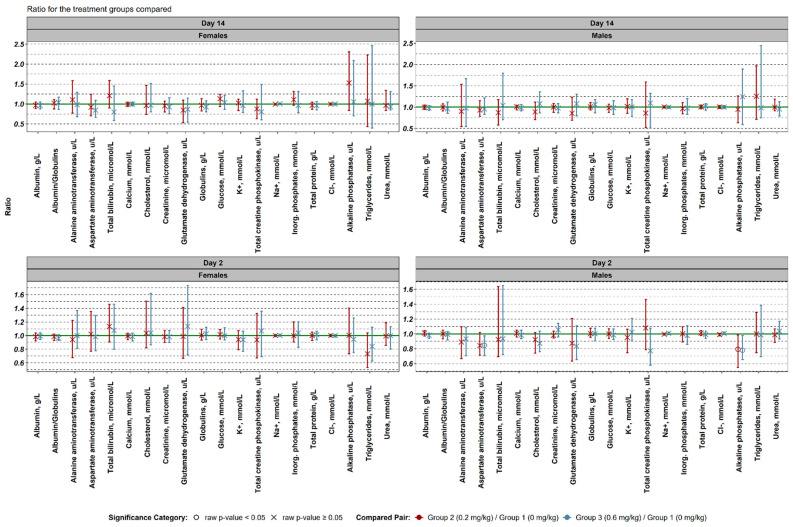
Comparison of the biochemical data between different experimental groups. Log10 transformed values were used to obtain median ratios and their non-parametric 95% confidence intervals. Reported values were backtransformed.

- Females and Males (Day 14):

No important alterations in biochemical parameters were detected in either sex. These results suggest that while the test compound causes some early, transient changes in liver-associated enzymes, particularly in males, the effects do not persist beyond the acute phase (Figure 4).

- Summary

A single dose of the experimental compound at 0.2 or 0.6 mg/kg was generally well tolerated in rats, with no mortality or overt clinical toxicity. Observed changes were primarily hematological, involving granulocyte, lymphocyte, and platelet dynamics, and transient biochemical alterations—especially in male liver enzymes. The findings underscore the need for further investigation into immune modulation and potential dose- and sex-specific responses in repeated-dose studies.

Postmortem autopsies of rats on days 2 and 14 after administration of the test drug did not reveal any pathomorphological changes in organs that can be considered toxicologically significant. During necropsy of males, isolated cases of discoloration of the thymus (focal or pitted, turning red) were observed 24 h and 14 days after treatment. Such cases were found in all groups, and pathomorphological examination confirmed hemorrhage of the thymic tissue. This anomaly may occur in experimental animals in isolated incidents, and in the absence of a higher incidence in groups taking the test drug, it could be linked to stressors.

**Figure 5:**
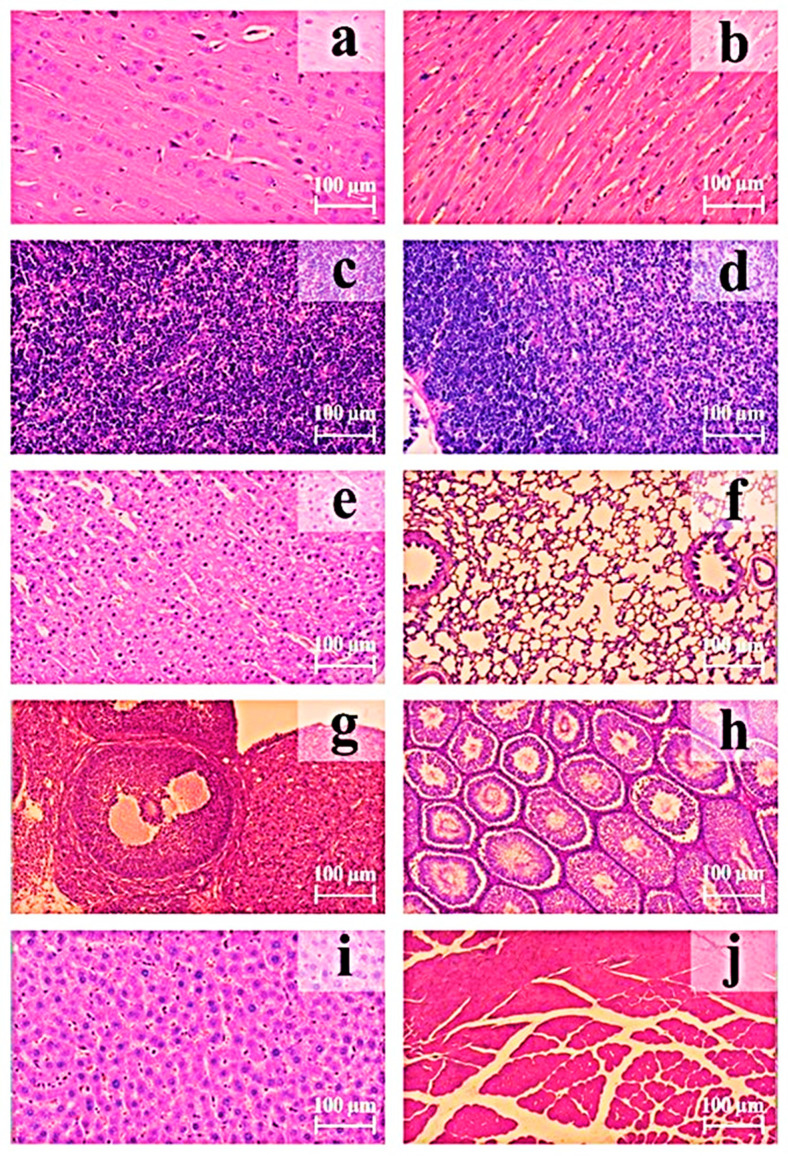
Histological samples of organs from rats in group 3 on day 2 after administration of pBudK-coVEGF-coANG + pBudK-coGDNF at 0.6 mg/kg. Hematoxylin–eosin staining. Hematoxylin stains cell nuclei blue to dark purple, eosin stains cytoplasm, extracellular matrix, and connective tissues pink to red. Abbreviations: (**a**) the cerebral cortex of the rat; (**b**) myocardium of the left ventricle of the rat; (**c**) mesenteric lymph nodes of the rat; (**d**) thymus of the rat; (**e**) adrenal cortex of the rat; (**f**) lungs of the rat; (**g**) ovary of the female rat; (**h**) testes of the male rat; (**i**) liver of the rat; and (**j**) musculus quadriceps femoris of the rat.

The histological results are presented in Figure 5.

Pathomorphological aspects were not observed in thigh muscle tissues at the injection site that would indicate local irritation or any other unfortunate side effect of the test drug. Mononuclear cell infiltration of the epimysium was observed in one female on day 2 after receiving the 0.6 mg/kg dose. However, this case was mild and isolated, making it difficult to determine whether it was related to the test drug or mechanical trauma from the injection needle.

In the immune organs (thymus, spleen, and lymph nodes), no significant differences were found in the number or severity of detected findings between the control and drug-administered groups. The indicators remained comparable in both groups. It is worth noting that the pathomorphologic findings identified are commonly observed in the control rat population and do not have toxicological significance.

Similarly, cases of glycogen accumulation in hepatocytes, manifestations of chronic progressive nephropathy, and mineralization in renal tubules, which were detected with a rather high frequency in the control population of rats, and their frequency and severity of manifestation did not increase after administration of the test drug.

No microscopic abnormalities were found in the liver that could be associated with decreased activity of the enzymes ALT, AST, and ALP in males on day 2 after administering the test drug.

In the adrenal glands of some females who received the test drug, cases of cytoplasmic vacuolization in cells of the cortical layer (particularly the zona fasciculata) were observed on the second and fourteenth days after administration, as opposed to the control group. These observations were not found in the control group. These cases may be likely related to a stronger immune response in females and a simultaneous generalized stress response to the injected drug, manifested by hematological effects. Cytoplasmic vacuolization in cells of the cortical layer is often associated with lipid accumulation under normal conditions and may reflect adaptive reactions related to steroid hormone synthesis (mainly corticosterone in rats) [17]. However, it is important to note that vacuolization was minimal and of mild severity and was observed only in individual females. In males, the observed occurrence of this result was not related to drug or solvent administration. Therefore, these results, within the range of biological variability, are not related to drug effects.

### 3.3. Analysis of Genotoxic and Mutagenic Effects

The studies showed that direct injection of the test drug had no effect on the clinical condition of the control and experimental animals. The animals in all experimental groups were active. They willingly consumed food and water throughout this experiment. No mortality was recorded in any of the groups.

The study of micronucleus test parameters showed that the drug in the doses studied (200, 1000, and 5000 μg/kg) had no genotoxic effect. The results are presented in Table 1. A notable increase in polychromatic erythrocytes with micronuclei was observed both in absolute and relative indices with the injection of cyclophosphamide. A total of 126–144 polychromatic erythrocytes with micronuclei per 4000 cells were recorded in the positive control group, which was 3.1–3.6% of the cells. In the negative control group and the groups where the drug was injected in the doses studied, this index was 10 times lower.

In the group of animals that received the gene therapy drug at doses of 200, 1000, and 5000 μg/kg, no increase in PCEs of micronuclei was recorded relative to the negative control group. All measured parameters of bone marrow erythroblasts after the use of the tested drug almost corresponded to the control group that received the solvent, water (Table 1).

Thus, the studies conducted showed that the gene therapy drug based on the plasmids pBudK-coVEGF-coANG and pBudK-coGDNF did not have a cytogenetic damaging effect. According to this test, this drug can be considered sufficiently safe according to the parameters of genotoxicity.

## 4. Discussion

This study provides novel evidence supporting the safety profile of a new non-viral gene therapy based on the dual plasmid system pBudK-coVEGF-coANG and pBudK-coGDNF, designed for intramuscular administration. Mice are commonly used laboratory animals for preliminary drug tests, including gene therapy. Animals of the same gender are adequate for the initial testing of tolerated doses [18]. For further toxicological studies, young reproductively mature male and female Sprague-Dawley rats (SD) were used as test subjects. This animal model is considered appropriate for toxicological research. The number of animals in the experimental groups is adequate to obtain reliable results during the acute phase after drug administration and in the phase following drug withdrawal [19].

Unlike viral vectors commonly used in gene therapy, which carry inherent risks of insertional mutagenesis and strong immunogenicity, this plasmid-based approach avoids integration into the host genome and elicits minimal immune response. This makes it particularly suitable for clinical applications requiring repeated or high-dose administration, such as the treatment of chronic ischemic or neurodegenerative conditions [20,21].

Our findings contribute new data to the existing body of research on plasmid-based gene therapies. Previous studies have shown that plasmid vectors can be safe and effective for transient gene expression, although few have bespoken a lack of toxicity at such extremely high doses—up to 1000 times the intended clinical dose—as shown here. Additionally, our results extend earlier findings by demonstrating the absence of both acute and delayed toxicological effects, including genotoxicity and mutagenicity, in multiple animal models [20,21].

One of the key observations was the presence of dose-dependent hematological changes, particularly in female animals, noted within 24 h of injection. While such effects could raise concern, they were transient, adaptive, and toxicologically insignificant, consistent with short-term immune modulation commonly seen following nucleic acid delivery. This aligns with reports from other non-viral gene delivery studies that describe temporary immune or hematologic shifts without long-term implications [22,23].

Interestingly, we observed a reduction in liver enzyme activity (AST, ALT, ALP, and GLDH) in male rats, which contrasts with the typical hepatic enzyme elevation seen in response to hepatocellular stress. While seemingly counterintuitive, this may reflect a temporary downregulation of hepatic metabolism in response to acute immunologic stimuli, rather than direct liver damage. Prior work has revealed that reductions in enzyme activity can occur in scenarios of metabolic adaptation or stress-induced hepatocellular quiescence [24,25,26]. Further mechanistic studies would help confirm this hypothesis.

Importantly, no histopathological abnormalities were found in major organs, supporting the conclusion that the observed biochemical and hematological changes were not linked to underlying tissue damage.

The micronucleus assay, a widely accepted genotoxicity test, confirmed the absence of mutagenic or chromosomal-damaging effects, further underscoring the safety of the plasmid combination. These findings are consistent with the expected safety of non-integrating DNA vectors but provide rare in vivo validation using clinically relevant doses.

In general, our data are consistent with important studies on the safety of gene therapy, which suggest that gene therapy, particularly plasmid therapy, is safe and can be used in the clinic [27,28].

- Limitations

Despite the promising results, this study has some limitations. First, the duration of observation was limited to the acute phase (24 h) and a 14-day recovery period, which may not fully capture potential long-term or cumulative toxic effects. Chronic administration studies would be needed to establish comprehensive safety for repeated dosing protocols.

Second, this study was conducted in healthy laboratory animals, which do not total replicate the complex immune or metabolic environments present in human disease conditions. The response in patients with underlying ischemia, neurodegeneration, or immune dysregulation could differ.

## 5. Conclusions

**In summary,** the findings of this study demonstrate that the novel non-viral gene therapy composed of plasmids **pBudK-coVEGF-coANG** and **pBudK-coGDNF** is safe and well tolerated in animal models, even at doses far exceeding the intended clinical level. The observed hematological and biochemical changes were transient, non-toxic, and likely represent adaptive physiological responses rather than indicators of organ damage. The absence of histopathological alterations, mutagenic effects, and delayed toxicity further supports the safety profile of this plasmid-based approach. While the results are promising, further long-term and disease-model studies are warranted to fully characterize the therapeutic window and clinical applicability of this gene therapy platform.

## Figures and Tables

**Table 1:** Summary data on the occurrence of micronuclei in bone marrow erythrocytes of mice after injection of the gene therapy plasmid drug.

	Group 1 (0 µg/kg)		Group 2 (200 µg/kg)		Group 3 (1000 µg/kg)		Group 4 (5000 µg/kg)		Group 5 (Cyclophosphamide 50 mg/kg)	
	MEAN ± SD	N	MEAN ± SD	N	MEAN ± SD	N	MEAN ± SD	N	MEAN ± SD	N
MALES										
Number of mature (normochromic) erythrocytes per 500 cells	237 ± 14	5	239 ± 10	5	248 ± 8		246 ± 1	5	246 ± 6	5
Number of polychromatic erythrocytes (PCEs) per 500 cells	263 ± 14	5	261 ± 9	5	252 ± 8		254 ± 1	5	254 ± 6	5
Share of PCEs in the total number of erythrocytes	0.53 ± 0.03	5	0.52 ± 0.02	5	0.50 ± 0.02		0.51 ± 0.00	5	0.51 ± 0.01	5
Number of PCEs with micronuclei per 4000 cells	12 ± 3	5	11 ± 1	5	12 ± 2		12 ± 4	5	144 ± 8 *	5
Number of PCEs with micronuclei, %	0.295 ± 0.082	5	0.285 ± 0.029	5	0.310±		0.300 ± 0.108	5	3.605 ± 0.206 *	5
FEMALES										
Number of mature (normochromic) erythrocytes per 500 cells	233 ± 13	5	233 ± 11	5	242 ± 15		237 ± 13	5	230 ± 28	5
Number of polychromatic erythrocytes (PCEs) per 500 cells	267 ± 13	5	267 ± 11	5	258 ± 15		263 ± 13	5	270 ± 28	5
Share of PCEs in the total number of erythrocytes	0.53 ± 0.03	5	0.53 ± 0.02	5	0.52 ± 0.03		0.53 ± 0.03	5	0.54 ± 0.06	5
Number of PCEs with micronuclei per 4000 cells	12 ± 4	5	10 ± 3	5	12 ± 1		14 ± 2	5	126 ± 26 *	5
Number of PCEs with micronuclei, %	0.305 ± 0.093	5	0.250 ± 0.071	5	0.305 ± 0.033		0.345 ± 0.054	5	3.160 ± 0.644 *	5

* *p* < 0.0001 relative to group 1 according to Kruskal–Wallis ANOVA test.

## Data Availability

The original contributions presented in this study are included in the article and Supplementary Materials. Further inquiries can be directed to the corresponding author.

## Supplementary Materials

The following supporting information can be downloaded at: https://www.mdpi.com/article/10.3390/biomedicines13051223/s1, Figure S1: Restriction analysis of the plasmid pBudK-coVegf-coAng and *pBudK-coGdnf*.
