# Supplementary material for: Extended Toxicity, Genotoxicity, and Mutagenicity of Combination of pBudK-coVEGF-coANG and pBudK-coGDNF Plasmids in Preclinical Trials
Source: Biomedicines. 2025 May 18;13(5):1223. doi: 10.3390/biomedicines13051223 (PMC12109478; doi:10.3390/biomedicines13051223)
Supplement: Supplementary file 1 [file biomedicines-13-01223-s001.zip › biomedicines-3572539-supplementary.pdf]

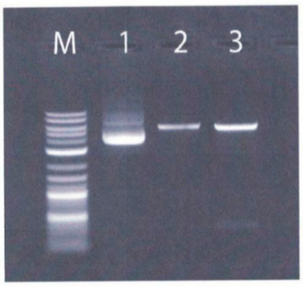

Restriction analysis of the plasmid pBudK-coVEGF-coANG. M - 1 kb Plus DNA ladder marker (NEB), 1 - plasmid DNA, 2 - linearized plasmid DNA (5,762 bp), 3 - Sacll restriction analysis (expected sizes: 5,431 bp and 331 bp).

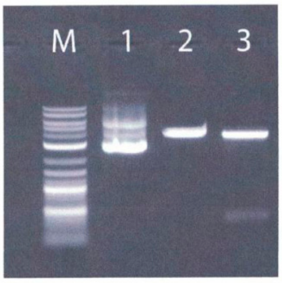

Restriction analysis of the plasmid pBudK-coGDNF. M - 1 kb Plus DNA ladder marker (NEB), 1 - plasmid DNA, 2 - linearized plasmid DNA (4,183 bp), 3 - Sacll restriction analysis (expected sizes: 3,852 bp and 331 bp).

Supplement Figure S1. Restriction analysis of the plasmid pBudK-coVEGF-coANG and pBudK-coGDNF
